# Short-Chain Fatty Acids as Bacterial Enterocytes and Therapeutic Target in Diabetes Mellitus Type 2

**DOI:** 10.3390/biomedicines11010072

**Published:** 2022-12-27

**Authors:** Maria-Adriana Neag, Anca-Elena Craciun, Andreea-Ioana Inceu, Diana-Elena Burlacu, Cristian-Ioan Craciun, Anca-Dana Buzoianu

**Affiliations:** 1 Department of Pharmacology, Toxicology and Clinical Pharmacology, Iuliu Hatieganu University of Medicine and Pharmacy, 400337 Cluj-Napoca, Romania; 2 Department of Diabetes and Nutrition Diseases, Iuliu Hatieganu University of Medicine and Pharmacy, 400006 Cluj-Napoca, Romania; 3 Faculty of Medicine, Iuliu Hatieganu University of Medicine and Pharmacy, 400012 Cluj-Napoca, Romania

**Keywords:** enteric nervous system, short-chain fatty acids, diabetes mellitus, gut microbiota, glucagon-like peptide 1

## Abstract

Diabetes mellitus is a disease with multiple gastrointestinal symptoms (diarrhea or constipation, abdominal pain, bloating) whose pathogenesis is multifactorial. The most important of these factors is the enteric nervous system, also known as the “second brain”; a part of the peripheral nervous system capable of functioning independently of the central nervous system. Modulation of the enteric nervous system can be done by short-chain fatty acids, which are bacterial metabolites of the intestinal microbiota. In addition, these acids provide multiple benefits in diabetes, particularly by stimulating glucagon-like peptide 1 and insulin secretion. However, it is not clear what type of nutraceuticals (probiotics, prebiotics, and alimentary supplements) can be used to increase the amount of short-chain fatty acids and achieve the beneficial effects in diabetes. Thus, even if several studies demonstrate that the gut microbiota modulates the activity of the ENS, and thus, may have a positive effect in diabetes, further studies are needed to underline this effect. This review outlines the most recent data regarding the involvement of SCFAs as a disease modifying agent in diabetes mellitus type 2. For an in-depth understanding of the modulation of gut dysbiosis with SCFAs in diabetes, we provide an overview of the interplay between gut microbiota and ENS.

## 1. Enteric Nervous System (ENS)

### 1.1. Introduction

Diabetes mellitus is a disease with multiple gastrointestinal symptoms (diarrhea or constipation, abdominal pain, bloating) whose pathogenesis is multifactorial [1]. The most important of these factors is the enteric nervous system (ENS), also known as the “second brain”; a part of the peripheral nervous system capable of functioning independently of the central nervous system [2,3].

ENS is a part of the peripheral nervous system contained in the intestinal wall that is able to function independently of the central nervous system (CNS), being organized into two plexuses: myenteric and submucosal; Auerbach’s and Meissner’s plexus, respectively [2,4,5]. ENS modulates multifold functions of the gastrointestinal tract to maintain intestinal homeostasis, such as intestinal permeability, immune function, gastrointestinal motility, or the maintenance of mucosal integrity [5].

Extrinsic and intrinsic factors may influence gene expression and ENS development and activity. ENS development may be affected by maternal nutrition and deficiency in vitamin A or folate or foetal exposure to neuromodulatory drugs (antidepressants, antipsychotics, anti-epileptics, anti-cholinergics) that change neurons’ activity and post-natal subtype and function [4]. 

The intestinal microbiota represents the main place of the body where the largest number of microorganisms live (bacteria, viruses, fungi, etc.), with five main bacterial phyla in healthy adults, represented by Firmicutes, Bacteroidetes, Actinobacteria, Proteobacteria, and Verrucomicrobia. The intestinal microbiota plays a decisive role in the homeostasis of the body and the maintenance of human health [6,7]. Depletion of compositional phila of gut microbiota have been evidence in a large spectrum of neurological diseases, i.e., stroke [8], neurodegenerative disorders, autism spectrum disorder [9], and multiple sclerosis [10], Parkinson’s disease, gliomas [11], cognitive disorders [12,13], and nervous system tumors [11].

Emerging experimental data suggests that depletion of gut microbiota with antibiotics results in impaired secretomotor function, as well as hypersensitivity of visceral pain-related responses, related to changes in ENS [14]. Multiple molecular features related to the disruption of the ENS–gut microbiota axis has been evidenced. In animal models with impaired gut microbiota, intestinal samples revealed immune changes, including increase of secretory-IgA, upregulation of lectin RegIIIγ, TLR-4 and 7, cannabinoid receptors 1 and 2, mu-opioid receptors, and nerve growth factor [14]. Moreover, in AB-treated mice, loss of nitrergic, cholinergic, and calretinin enteric neurons of the myenteric and submucosal plexus, along with neuronal fiber density have been evidenced [15]. 

Modulation of the ENS with Short chain fatty acids (SCFAs) represents a potential therapeutic target in diabetes. The role of microbiota-derived metabolites, such as lipopolysaccharides (LPS) and SCFAs in neuron survival and neurogenesis has been evidenced in experimental AB-induced gut dysbiosis [15]. 

Much scientific interest is focused on improving insulin delivery technologies based on well-designed polymers to avoid adverse effects associated with the subcutaneous route of insulin administration, i.e., pain, lipodystrophy, and hyperinsulinemia [16]. However, an equal effort should be directed toward adjuvant natural therapeutic agents that could modulate gut microbiota in diabetes patients.

Gut microbiota, via SCFAs, may play a role in the regulation of blood pressure, glycemia, coagulability, and lipid profile during pregnancy and may also impact newborns’ health via the vertical transfer of microbiota and (or) its metabolites [17]. By modulating immune response, SCFAs could provide a potential beneficial effect for patients with diabetes [18]. 

Multiple nutraceuticals (probiotics, prebiotics, and alimentary supplements) have been proposed as therapeutic agents aimed to increase the amount of short-chain fatty acids and achieve the beneficial effects in diabetes [18]. 

This review outlines the most recent data regarding the involvement of SCFAs as a disease-modifying agent in diabetes mellitus type 2. For an in-depth understanding of the modulation of gut dysbiosis with SCFAs in diabetes, we provide an overview of the interplay between gut microbiota and ENS.

### 1.2. ENS Overview

The enteric nervous system (ENS) is a part of the peripheral nervous system (PNS), contained in the intestinal wall, that is able to function independently of the central nervous system (CNS) [2,3]. The ENS, organized into two plexuses (myenteric and submucosal; Auerbach’s and Meissner’s plexus, respectively), is a complex network containing excitatory and inhibitory interneurons, motor neurons, glial cells, intrinsic primary afferent neurons (IPANs, sensory), and interstitial cells of Cajal (ICC) [2,4,5]. The complex network of neurons forming ENS may regulate enteric behavior independently of the CNS; even though there is a bidirectional link between these two systems, the ENS and the CNS, under normal conditions [19]. ENS is derived mainly from the neural crest that contains vagal, truncal, and sacral neural crest cells. The development of ENS relies on glial cell line-derived neurotrophic factor (GDNF), a member of the TGFβ superfamily, that is involved in guiding the progenitor cells, promoting the proliferation and differentiation of enteric neural crest-derived cells (ENCCs) and establishing the number of neurons in adulthood [2].

### 1.3. The Organization of ENS

**Figure 1:**
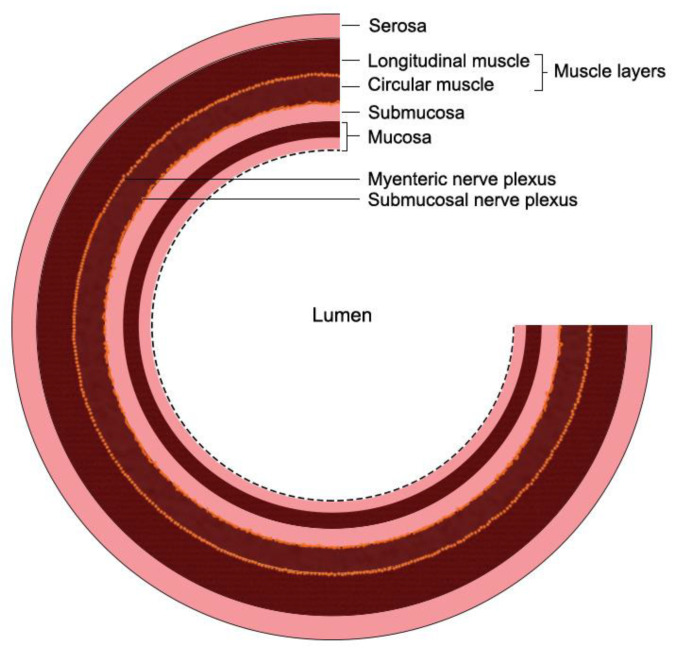
The organization of gut wall layers.

The gut wall comprises four layers, the mucosal layer with a mucosal barrier, submucosal, muscular, and serosa layers. The mucosal barrier is composed of the mucin layer, the epithelial cell layer, and the apical junction complex [3]. ENS consists of the submucosal plexus situated beyond the epithelial cell layer and the myenteric plexus localized between the longitudinal and circular muscle layers. These two layers are connected to each other through neuronal projections that also connect to non-neuronal targets, such as immune cells and epithelial cells. The first ENS components that are close to the gut lumen are glial cells [4]. A short overview of gut wall layers is provided in Figure 1. 

Enteric glial cells (EGCs) are defined by all peripheral neuroglia that are associated with the enteric neurons. Their main function is to assure local homeostasis using bidirectional communication. Based on their location in the intestinal wall, six main types of EGCs have been described. Intraganglionic glial cells are associated with neuronal bodies in the myenteric and submucosal plexus; the submucosal glia cells mediate the secretomotor function of the submucosal neurons, whereas the myenteric glial cells are involved in myenteric neurons trophic support, modulation of oxidative stress and neuroinflammation, neurogenesis and gliogenesis. Interganglionic glial cells surround the nerve bundles and mediate the signal propagation between myenteric ganglia. Extraganglionic glial cells include glia associated with nerve fibers in the two plexuses, but outside the ganglia, glia localized in the intestinal mucosa, and glia associated with nerve fibers in the muscle layer. The mucosal glial cells are involved in epithelial cell maturation and have potential roles in the modulation of immune reaction and neuroendocrine signalling [5]. 

IPANs are key components of the ENS that modulate enteric activity and assure adequate bowel function. IPANs consist of Dogiel type I and type II morphology neurons, cholinergic myenteric neurons, nitrergic myenteric neurons, and intestinofugal neurons. Dogiel type I morphology neurons were described as responsive to mechanical stimuli, but with rare ramifications to the mucosa. Dogiel type II morphology neurons consist of multipolar processes that ramify extensively in the mucosa and circumferentially and have both mechanosensory and chemosensory properties. Cholinergic and nitrergic myenteric neurons were proven to be rapidly adapting excitatory neurons. Intestinofungal neurons connect with sympathetic neurons from the prevertebral ganglia, being stimulated by mechanical compression. They have been involved in the increase of sympathetic inhibition of gut motility [19].

### 1.4. The Functions of ENS

The myenteric plexus is responsible for intestinal muscle movements related to gut content propulsion, whereas the submucosal plexus coordinates the secretion and absorption of biomolecules. ENS modulates multifold functions of the gastrointestinal tract to maintain intestinal homeostasis, such as intestinal permeability, immune function, gastrointestinal motility, or the maintenance of mucosal integrity [5]. 

The ENS or intrinsic nervous system represents an important mechanism in the communication between the gut and the brain, along with neurotransmitters, i.e., acetylcholine, serotonin, and dopamine produced by the gut microbiota and enteroendocrine cells of the gut [19,20]. ENS proceeds key regulatory functions to maintain intestinal homeostasis, from motor functions, enteric transport and secretion, local blood flow regulation, to immune and endocrine responses [21]. The enteric neural circuits which form ENS are located in two types of ganglia, including Auerbach’s plexus or myenteric plexus, responsible for motor control of circular and longitudinal muscle layer and Meissner’s plexuses, consisting of enteric neurons which innervate epithelial and the smooth muscle layer of the muscularis mucosae [21,22].

The propulsion of content along the gut requires ENS activity, whereas phasic contractions are not dependent on enteric neurons. ICCs, the gut pacemaker cells, are involved in the generation of electrical oscillations in the smooth muscle cells from the intestinal wall that cause phasic contractions [19]. In a genetic study based on loss of function of ICCs, the authors showed that ICCs integrate excitatory and inhibitory signals from enteric neurons, with slow-wave activity of the smooth muscle cells, indicating that ICCs have an important role in the coordination and control of gut motility beyond their role of pacemaker cells [20]. 

The myenteric neurons connect with SIP syncytium, an essential key factor for bowel motility. The SIP syncytium is a multicellular syncytium formed of smooth muscle cells (SMCs), ICCs, and platelet-derived growth factor receptor (PDGFR) α cells connected through gap junctions. The SIP name is derived from the first letter of each component. Bowel motility is based on motility patterns, like peristalsis, segmentation, and migrating motor complex in the small intestine and high amplitude propagating contractions (HAPCs) in the colon. Activation of the SIP syncytium through diverse neural signalling generates and maintains these motor patterns [21]. ENS motility patterns include chemo-transduction that relies on endogenous 5-HT and chemoreceptor activation of Dogiel type II morphology neurons, and neurotransmitters mediation, which involve acetylcholine action over nicotinic receptors, glutamate signalling, ATP/P2X pathway and vasoactive intestinal peptide signalling [19]. 

Spencer et al. showed that rhythmic electrical depolarizations in smooth muscle cells are based on rhythmic firing in ENS that are responsible for colonic migrating motor complexes (CMMCs). Temporally synchronized neurons, both excitatory and inhibitory, were activated at the onset of neurogenic contractions. ENS was activated before smooth muscle cell depolarization, with a rhythmic and temporally synchronized firing pattern at a frequency around 2 Hz, indicating the discovery of a unique motility pattern outside the CNS [22]. 

The enteric circuit relies on inputs represented by the intrinsic sensory pathways and extrinsic mediation. Intrinsic sensory pathways include the detection of chemical and mechanical stimuli and the feedback generated by the contracting muscles. Although intrinsic sensory neurons form an extensive network, the nerve endings are not in direct contact with gut content. Enteroendocrine cells (EECs) found in the epithelium lining are stimulated by the nutrient composition of the luminal content, and secrete many peptides and hormones involved in paracrine or endocrine mechanisms and in communication with ENS. The communication between EECs and ENS includes the release of 5-HT from EECs and the activation of 5-HT3 receptors from the intrinsic sensory nerve endings [23]. Sympathetic and vagal afferent nerves are responsible for extrinsic mediation based on cholinergic and adrenergic signalling, defining the complex relationship between CNS and ENS [23]. 

ENS-mediated vasodilation is mediated by the stimulation of IPANs by the released 5-HT induced by small distortions of the mucosa, and the activation of mechanosensitive enteric neurons as a response to mechanical deformations of the gut [24]. The common pathway is the activation of muscarinic M3 receptors by the acetylcholine in endothelial cells, which leads to NO secretion and vasodilation. Other mediators involved in ENS-mediated vasodilation include substance P, VIP, or histamine [21]. 

Fluid and electrolytes secretion into the lumen also relies on IPANs activation by 5-HT and further release of acetylcholine or VIP. These mediators act via their corresponding receptors (muscarinic, respectively, VIPR1) and increase intracellular calcium and cyclic AMP, that activate chloride channels, inducing the transport of chloride and subsequently accompanying sodium and water [21]. 

Other functions of the ENS are the regulation of epithelial proliferation, differentiation and repair and the modulation of epithelial barrier [21]. An overview of the main functions of the ENS and their influencing factors is provided in Table 1.

### 1.5. Factors That Influence ENS Activity

Extrinsic and intrinsic factors may influence gene expression and ENS development and activity. ENS development may be affected by maternal nutrition and deficiency in vitamin A or folate or foetal exposure to neuromodulatory drugs (antidepressants, antipsychotics, anti-epileptics, anti-cholinergics) that change neurons activity and post-natal subtype and function [4,26]. 

After birth, the diet’s composition may directly impact ENS activity or may change gut microbiota composition and affect secondarily ENS. The nutrients involved in ENS mediation have been proposed to be SCFA butyrate that can alter neuron activity and gene expression and N-3 polyunsaturated fatty acids that can affect enteric neuron subtype ratios. Breast milk, enriched in nutrients and bioactive molecules, including neurotrophic factors and cytokines, has been involved in the modulation of enteric neurons and glial cells, particularly during the first postnatal stages [4]. Nezami et al. have been shown in mice fed with a diet with 60% calories from fat, delayed intestinal transit, associated with myenteric neurons cytoplasmic lipid accumulation and apoptosis, mitochondrial dysfunction, and endoplasmic reticulum stress [25]. A low-palmitate high fat diet did not cause similar changes, suggesting that palmitic acid was responsible for enteric neuronal cell dysfunction. They also showed that the effects were mediated by miR375 overexpression in association with reduced levels of 3-phosphoinositide-dependent protein kinase-1 (Pdk1), both markers of cell survival, differentiation, and apoptosis regulators [25]. Furthermore, vitamin D supplementation protected against myenteric neurons dysfunction induced by high fat diet and palmitic acid. The suggested mechanism was that vitamin D induces activation of peroxisome proliferator-activated receptor gamma (PPAR-γ), leading to ameliorated neuronal peroxisome function and improved neuronal lipid metabolism [26]. 

Treatment with antibiotics can also influence ENS activity. The administration of vancomycin in the first post-natal 10 days in mice changed the level of activity of enteric neurons in young adult life depending on the sex and decreased the level of mucosal 5-HT on the long term. In male mice, vancomycin caused a reduction of neurons and glial cells in the myenteric plexus and altered the subtype proportions of neurons. The excitability of myenteric neurons was also decreased in male mice after vancomycin treatment. In female mice, there were no alterations regarding myenteric neurons, but the submucosal neurons were more responsive to stimuli. This study suggests that neonatal exposure to antibiotics can change the function of ENS later in life, leading to gastrointestinal motility disorders, depending on the sex [27]. 

CNS disorders can also have an impact over ENS function. In a mice model of permanent middle cerebral artery occlusion (pMCAO), the authors concluded that in focal ischemic stroke, enteric neuronal apoptosis is caused by a mechanism involving galectin-3 release triggered both central and peripheral and further activation of toll-like receptor 4 (TLR-4) and transforming growth factor- β- activated kinase 1 (TAK1)/AMP activated kinase (AMPK) pathway, suggesting a neuroinflammatory reaction transmitted from CNS to ENS [28]. 

## 2. The Interplay between ENS and Gut Microbiota

The intestinal microbiota represents the main place of the body where the largest number of microorganisms live (bacteria, viruses, fungi, etc.) [6]. There are five main bacterial phyla in healthy adults: Firmicutes, Bacteroidetes, Actinobacteria, Proteobacteria, and Verrucomicrobia [6,7]. The intestinal microbiota plays a decisive role in the homeostasis of the body and the maintenance of human health [31]. Depletion of compositional phila of gut microbiota have been evidence in large spectrum of neurological diseases, i.e., stroke [8], neurodegenerative disorders, autism spectrum disorder [9], and multiple sclerosis [10], Parkinson’s disease, gliomas [11], cognitive disorders [12,13], and nervous system tumors [11]. Evolving experimental studies explored the relation between dysbiosis induced by antibiotics and changes in microbiota and gut–brain axis signaling [14,32]. In mice model, treatment with high doses of Antibiotics (AB) alters gut microbiota, with enrichment in Enterobacteria, and decreases in Bacteroides and Enterococci populations [32]. Regarding enteric neuron circuits within the intestinal layers, long-term AB usage in mice increases MPO and SP immunoreactivity in the area of submucous and myenteric plexus resulting in visceral hypersensitivity to colorectal distension [32]. This further suggests the involvement of decreased gut bacterial species induced by AB usage in disruption of homeostasis of ENS. Targeting gut microbiota disruption with pro/prebiotics could restore the gut microflora and therefore reestablish ENS homeostasis in AB-treated mice. Compositional microflora changes in AB-treated mice were represented of enrichment in *Bacteroides* spp., Clostridium coccoides, *Lactobacillus* spp. and underrepresentation of *Bifidobacterium* spp. [32]. After AB deprivation of beneficial species of gut microbiota, mice treated with Lactobacillus paracasei shown reduced visceral hypersensitivity and SP immunostaining compared with controls [14]. Local epithelial and immune cells express Toll-like receptors (TLRs), which recognize bacterial ligands, called Microbial-associated molecular patterns (MAMPs), and bacterial-derived metabolites, inducing specific local and systemic immune responses [33,34]. Recognition of MAMPs by TLR represents the basis of the interaction of gut microbiota with ENS.

### 2.1. Crosstalk between Enteric Neurons and Gut Microbiota

Gut dysbiosis causes alteration in host microbial interactions of IBS patients, leading to dysregulated local immune response, which might contribute to intestinal sensorial and secretomotor disruption in these patients [14]. Neuronally-dependent intestinal changes have been shown in animal studies with disrupted gut microbiota, which affected colonic motility, pain related visceral sensitivity, and secretomotor function [14]. Multiple immunological and neuronal expression theories of the ENS might explain intestinal changes during gut dysbiosis.

Evolving experimental data evidenced the role of the TLR-signalling pathway in regulating neuronal survival and neurogenesis of neural plexuses of intestines [35,36,37,38]. Immunostaining studies showed differential expression of TLR on nitrergic, cholinergic and calretinin neuron populations of myenteric and submucosal neurons [15,35,36]. In mice with TLR2 deficiency and gut dysbiosis, ENS exhibited changes in architecture and neuromodulator profile, with alteration of secreto-motor function, and reduced glial cell line-derived neurotrophic factor (GDNF) levels in smooth muscle cells. These changes were reversed by administrating TLR2 agonist [37].

In normal conditions, TLRs are constitutively expressed on surface of microglia and glial cells, with activation of TLR pathway in disease conditions [39,40]. TLR are constitutively expressed in glial cells and microglia under normal conditions, but changes in TLR expression in neuronal cells have been observed under pathological conditions [38]. However, in C57BL/6N specific pathogen free (SPF) mice, the expression of TLR3, TLR4, and TLR7 has been noted in nerve fibers and ganglia of myenteric and submucous plexuses of the small and large intestine [38]. Moreover, dorsal root ganglia of thoracic and lumbosacral areas in SPF-mice exhibited differential expression of TLR3, TLR4, and TLR7, suggesting the role of TLR signaling in ENS regulation [38]. 

In mice studies, AB treatment affect intestinal integrity leading to IBS-related mechanisms, such as disruption of gastrointestinal motility, hypersensitivity of visceral pain-related responses, enhanced secretomotor function. Using chemical analysis methods in animal models with impaired gut microbiota, intestinal samples revealed immune changes, including increase of secretory-IgA, upregulation of lectin RegIIIγ, TLR-4 and 7, cannabinoid receptors 1 and 2, mu-opioid receptors, and nerve growth factor [14].

Vicentini et al. showed a loss of nitrergic, cholinergic, and calretinin enteric neurons of myenteric and submucosal plexus in AB-treated mice, along with neuronal fiber density [15]. Notably, enteric neurogenesis, suggested by upregulation of Sox2 in neurons, accompanied dysregulated gut microbiota induced by AB in mice, suggesting the compensatory response triggered by ENS to gut microbial depletion after AB treatment [15]. Moreover, the recovery of gut microbiota was associated with restoration of intestinal epithelial barrier function and increase of enteric glia and neurons by stimulating enteric neurogenesis [15].

In mice treated with AB, dysregulated gut microbiota triggered enteric neuron changes within submucosal and myenteric plexuses. The enteric neuron changes exhibited a loss of neuronal population in nitrergic and cholinergic subpopulation, with a decrease of neuronal fiber density [15].

These findings outline that disruption in gut microbiota species triggers complex immune responses, which induces neurosensorial and neuroimmune modulators responsible for the loss of enteric neuron structure and chemical signals of ENS neurotransmitters [15]. 

### 2.2. The Role of SCFAs in ENS Regulation 

Short-chain fatty acids (SCFAs): acetate (C2), propionate (C3), and butyrate (C4) are the major end products of dietary fiber and starch metabolism by gut microbiota (microbial fermentation) [41]. C2 results under the action of *Lactobacillus* spp., *Bifidobacterium* spp., *Akkermansia muciniphila*, *Blautia hydrogenotrophica*, *Bacteroides* spp., *Ruminococcus* spp., C3 under the action of *Bacteroidetes or Firmicutes*, and C4 under the action of Firmicutes (*Ruminococcaceae and Lachnospiraceae*) [41,42].

SCFA serve as the primary energy source for colonocytes (especially butyrate) and contribute to the maintenance of intestinal barrier integrity through mucus production and increased expression of tight junction proteins (occludins, claudins, and junctional adhesion molecules) [43]. The highest concentration of SCFA was determined in the colon and the ratio of C2, C3, and C4 was 60:20:20, but lower concentrations of SCFA were identified in the liver and blood. In this regard, it can be mentioned that the main role of SCFAs begins in the intestine [41].

The link between SCFAs and the ENS is based on the interaction of SCFAs with their receptors, which are highly expressed in the ENS [44]. The first receptors identified as SCFA receptors were FFAR2 (GPR43) and FFAR3 (GPR41). The activation of these receptors influences several physiological processes (inflammatory, immune, hormonal secretion, etc.) contributing to maintaining the body’s homeostasis [45]. Another receptor responsible for SCFA effects is GPR109a. The interaction between this receptor and SCFA (C4) is important for intestinal homeostasis [46].

Through its primary mediators, SCFAs including acetate, propionate, and butyrate, the gut microbiota exerts immunomodulatory and anti-inflammatory functions and interacts in a bidirectional manner with numerous organ systems, forming the so called specific gut-microbiota-related axis, between microbiota and other organs [47,48]. Recent studies described so far, the Gut–Liver axis [49], Gut–Brain axis [50], Gut–Liver–Brain axis [51].

The role of microbiota-derived metabolites, such as lipopolysaccharides (LPS) and SCFAs in neuron survival, and neurogenesis has been evidenced in experimental AB-induced gut dysbiosis [15]. LPS supplementation during treatment with AB-attenuated nitrergic neuron loss in submucous and myenteric plexuses in mice [15]. SCFAs supplementation in AB-induced microbial depletion in mice dampens neuronal deficit in ileal and colonic myenteric plexus, by regulating S100B+ expression of neurons and enteric glial cells [15]. However, LPS and SCFAs did not succeed to restore impaired motor function and disrupted permeability of colon after AB treatment, suggesting long-term structural and microbial changes within intestine during AB treatment.

5-hydroxytryptamine (5-HT) represents a key factor that regulates secreto-motor function of the GI tract by acting on specific receptors localized on enterocytes, enteric neurons, and immune cells [52,53].

According to multiple studies, disruption in intestinal motility has been correlated with reduced 5-HT values within the gut [52,53]. 

SCFAs could regulate intestinal dysmotility by increasing tryptophan hydroxylase 1 expression in mice colonized with human gut microbiota, compared to SPF mice [53]. SCFAs modulate colonic serotonin production by acting on enterochromaffin cells [53]. Research data suggested that SCFAs might promote neurogenesis in gut dysbiosis by activating enteric serotonin networks via 5-HT4 receptors [53,54,55]. The amount of SCFA lowers the pH in the colon. Consequently, the microbiota at this level may be affected and subsequently SCFA production. These changes can influence the aggregation of proteins that then play an important role in blood sugar regulation. One of these proteins is IAPP secreted by pancreatic β cells [56,57].

**Figure 2:**
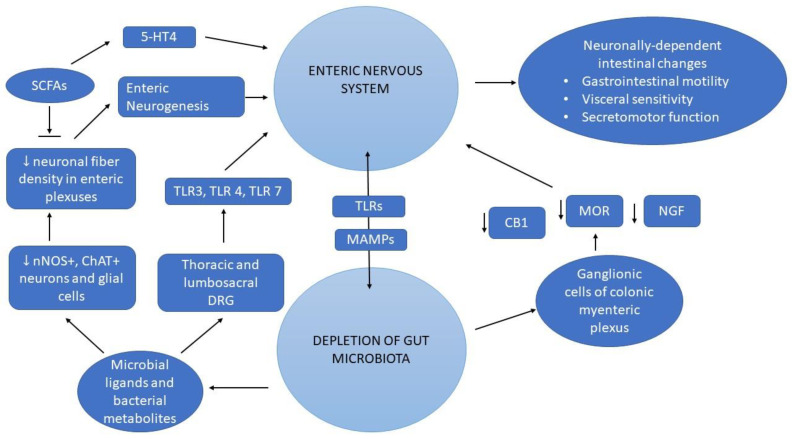
Common pathways shared between gut microbiota and Enteric Nervous System. Abbreviations: CB1, cannabinoid receptor 1; ChAT+, Choline acetyltransferase; DRG, dorsal root ganglia; SCFAs, 5-HT4, 5-Hydroxytryptamine receptor 4; MAMP, microbe-associated molecular pattern; MOR, mu-opioid receptors; NGF, nerve growth factor; nNOS+, Neuronal nitric oxides synthase; SCFAs, Short-chain fatty acids; TLRs, Toll-like receptors.

An overview of the main mechanisms shared between gut microbiota and ENS are provided in Figure 2.

## 3. The Link between SCFA and Diabetes Mellitus

It is estimated that 1 in 10 adults has diabetes mellitus (DM) and the estimated number of people living with diabetes in 2021 was around 537 million [58]. A healthy diet, with increased consumption of prebiotics, soluble fiber (such as pectin, inulin, and hemicellulose contained in fruits: apples, apricots, oranges, peaches, figs, and pears; vegetables: peas, turnips, sweet potatoes, and brussels sprouts; legumes: lima, navy, pinto, kidney, and black beans and in bran cereals, oatmeal, barley) and resistant starch, (from whole grains including oats and barley, brown rice, green bananas, white beans, and lentils), added to the other components of a healthy lifestyle, is well known to have beneficial effect in all types of DM, being the basement of clinical management [59].

The link between gut microbiome–SCFAs–complex diseases as DM is confirmed by microbiome-wide association studies (as association) and also by bidirectional Mendelian-randomization study (as causation).

Meta-analysis of gut microbiome studies showed that obesity and other obesity related diseases (such as T2DM) are associated with a loss of bacteria that produce C4 [60]. The causal relationship between the gut microbiome and glucose control was demonstrated by Sanna et al. [61], using data collected from 952 normoglycemic individuals (LifeLines-DEEP cohort) including genome-wide genotyping, gut metagenomic sequence and fecal SCFAs levels, combined with seventeen metabolic and anthropometric traits and then performing Mendelian-randomization of three genetic predictors effects in the discovery dataset (DIAGRAM: 26,676 T2DM cases and 13,2532 controls) and in the replication cohort (UK Biobank: 19,119 T2DM cases and 423,698 controls). They found that genetic predisposition to increased fecal concentration of propionate (C3) (meaning altered intestinal absorption or altered production?) is causally related to increased risk of T2DM. On the other hand, increased (genetic-driven) production of intestinal butyrate (C4) was associated with better insulin response following an oral glucose tolerance test.

Recent data showed that SCFAs could also play an important role in enhancing gut barrier function, preventing inflammatory clinical conditions associated with invading bacteria from the intestine. SCFAs deficiency may affect the gut–neuro–immune crosstalk, with important contributions to early stages of impaired autoimmunity response in type 1 diabetes mellitus (T1DM) [62].

The link between human microbiome–SCFAs (as potential therapeutic agents)–gestational diabetes mellitus (GDM) is also investigated. A review that included 128 studies concluded that gut microbiota, via SCFAs, may have a role in the regulation of blood pressure, glycemia, coagulability, and lipid profile during pregnancy, and may also impact on newborn health via vertical transfer of microbiota and (or) its metabolites. A major limitation of this review is that most of the observations were from animal models and the translation of the results to humans is difficult to relate [17].

In a recent published study that enrolled 60 pregnant women (40 controls and 20 GDM cases) showed that the levels of three dominant SCFAs (C2, C3, and C4) and total SCFAs were decreased in GDM compared to controls [63]. It was also observed in the same study that in pregnancies complicated with GDM, the placental content of specific receptors of SCFAs was decreased, associated with increased inflammatory responses. The study participants were further divided into four subgroups: first—normal pregnancies; second—GDM with isolated basal hyperglycemia; third—GDM with normal fasting plasma glucose but impaired result in 1 h and/or 2 h glycemia post glucose load; fourth—GDM with elevated fasting plasma glucose and/or 1 h and/or 2 h glycemia post glucose load. C3 levels were significantly decreased in the third group. The circulating levels of C2, C4, and total SCFAs were significantly reduced in the fourth group. After stratification according to BMI, only C2 was significantly lower in the overweight/obese GDM cases from the fourth group compared to the normal-weight control group. 

### 3.1. Short-Chain Fatty Acids as Therapeutic Target in Diabetes Mellitus

Gut homeostasis represents a microbial balance state maintained by local and system immune host functions, influenced by intestinal environmental factors. Complex interaction with multiple cells concur at gut homeostasis, such as innate immune cells, dendritic cells, macrophages and innate lymphoid cells, adaptive immune cells, T cells, B cells and plasma cells, and intestinal epithelial cells [64].

SCFAs produced by fermentation in the intestinal lumen play an important role in gut homeostasis, but they are also absorbed and reach blood circulation, with effect on glucose storage in the liver, muscle, and fat tissues, and direct effect on beta-cells of the pancreas. Being small molecules, they also reach the brain, with beneficial effect on appetite control and decrease food consumption, with consecutive weight loss and decrease of insulin resistance, having the potential to improve glycemic control of patients with diabetes, especially T2DM. There is evidence that SCFAs can have multiple beneficial effects in DM by stimulating glucagonlike peptide-1 (GLP-1) and insulin secretion, anti-inflammatory properties, anti-obesogenic effect, and improvement of insulin-sensitivity [18].

Recent findings indicate that SCFAs can also play a role in modulating immune response, with a potential beneficial effect for patients with T1DM. There are also few data regarding the role of SCFAs in gestational diabetes. 

Human cells express specific receptors for SCFAs, such as free-fatty acid receptor 2–FFAR2 (GPR43), which demonstrated highest selectivity for C2 (but can be also activated by other SCFAs) and free-fatty acid receptor 2–FFAR3 (GPR41), which can be also activated by SCFAs (pentanoate was the most potent agonist). Regarding the ligand preference of C2, C3 and C4, data showed for FFAR2 that C2 = C3 > C4 and for FFAR3 that C3 > C4 > C2 [64]. The adipose tissue had the highest expression of FFAR3 (on the endothelial cells of the blood vessels), but was also present in immune cells or endothelial cells in other tissues, while FFAR2 was most abundant in immune cells [65].

One potential SCFA as therapeutic agent and with evidence for beneficial results on human health is acetate (C2). However, there are different effects for oral administration, colonic or parenteral C2 infusion, acetogenic probiotic administration, or increasing acetogenic fiber consumption [66].

Early suggestions about the potential therapeutic effects of C2 come from folk medicine, where vinegar (which contains mainly C2) is used for its antihyperglycemic activity. In 2004, Johnston CS et al. published in Diabetes Care a study entitled “Vinegar Improves Insulin Sensitivity to a High-Carbohydrate Meal in Subjects With Insulin Resistance or Type 2 Diabetes” [67]. In this study, eight non-diabetic, insulin-sensitive control subjects, 11 non-diabetic insulin-resistant subjects, and 12 subjects with T2DM were enrolled. Fasting administration of a solution (containing 20 g of apple cider vinegar, 40 g of water and 1 tsp saccharine) compared to placebo, 2 min before the ingestion of a test meal (containing 87 g of carbohydrates), raised whole-body insulin sensitivity in the first hour of postprandial condition by 34% in insulin-resistant subjects and by 19% in T2DM subjects. The potential mechanism may be explained by the suppressing effect of C2 on disaccharidase activity [68] and by the rising effect on glucose-6-phosphate concentrations in skeletal muscle [69].

In another study, published by Halima et al., 46 T2DM patients were randomly assigned to receive either 15 mL of apple cider vinegar before the middle meal (active group, *n* = 24) or water (placebo group, *n* = 20) for 30 days. At the end of the study, significant reductions of the fasting plasma glucose, weight and body mass index (BMI), triglycerides and VLDL-cholesterol particles were observed in the active group, with no significant change of the parameters in the placebo group [70].

Another study, with the same design (15 mL of vinegar before the middle meal versus placebo, for one month) with 30 T2DM subjects in the active group versus 30 T2DM subjects in the placebo group, confirmed a beneficial effect of apple cider vinegar in the reduction of FPG and HbA1c, with no change in the placebo group. After one month of intervention, mean FPG levels decreased from 174.67 ± 63.52 mg/dL to 156.23 ± 60.04 and HbA1c from 7.56 ± 3.01% to 7.03 ± 2.97% [71].

In a group of 55 T2DM patients with poor glycemic control (mean HbA1c at the beginning of the trial was 9.32 ± 1.74%) the consumption of 15 mL of apple cider vinegar in 200 mL of water during dinner for three months significantly improved the glycemic parameters: FPG decreased from 170.14 ± 62.42 mg/dL to 157.32 ± 58.16 mg/dL and HbA1c at the end of the trial was 8.65 ± 1.81% [72].

However, as a conclusion about the effect of apple cider vinegar on glucose parameters in T2DM, a recent published systematic review and meta-analysis including nine randomized, placebo-controlled clinical trials published until January 2020, showed that the reduction of FPG was non-significant. After stratification based on the duration of intervention, the use of apple cider vinegar has a lowering effect on FPG in studies that lasted more than 8 weeks, but no dose-dependent effects were observed. The lowering effect of apple cider vinegar on HbA1c was non-significant in all groups [73].

A study comparing the acute effects of intravenous or intrarectal administration of C2 in six females with hyperinsulinemia showed that rectal administration was followed by increased peptide YY and GLP-1 release compared to intravenous administration or placebo. In this study, C2 administration increased plasma levels of peptide YY, GLP-1 and decreased the levels of TNF-alpha compared to placebo, with no change on plasma adiponectin [74]. 

Acute oral (but not intravenous) C4 administration in mice decreased caloric intake. Chronic C4 administration also prevented diet-induced obesity and activates brown adipose tissue (effects that are lost after subdiaphragmatic vagotomy), so C4 may play a role in preventing/treating obesity and obesity-related diseases, such as T2DM, in humans [75]. The role of C4 supplementation in modulating inflammation was studied in 37 subjects (18 healthy controls and 19 T2DM patients). There was significant difference between controls and T2DM patients regarding TNF-alpha and IL-10 circulating levels. C4 appears to suppress TNF-alpha production and increase IL10 levels. It also decreases the migration of monocytes in T2DM, with potential beneficial effects in the modulation of inflammation in this clinical condition [76]. In a 90-days controlled, open-label study that enrolled 16 T2DM patients randomly allocated 1:1 to diet alone or diet plus fecal microbiota transplantation, both methods showed improvement in glycemic (measured as HbA1c) and blood pressure control and weight loss. It was observed a change in the structure of microbiota that can suggest increase production of C4, but blood or colonic levels of SCFAs were not directly measured [77].

Both FFAR2 and FFAR3 are expressed in enteroendocrine cells and in vitro studies showed that C2 can increase, in a dose dependent manner (3.0 to 30 mM), the expression of proglucagon (a GLP-1 precursor) [78]. SCFAs can increase the GLP-1 release from the intestinal L-cells and the mechanism of action seems to be mediated through specific receptors–FFAR2 and FFAR3, followed by a rise of intracellular calcium, as previously demonstrated on mice colonic cultures. The release of GLP-1 is higher after incubation for 2 h with 1 mmol/L C3 or C2, with the smallest effect after incubation with 1 mmol/L of C4 [79].

The administration of 24 g inulin increased SCFAs areas under de curve (AUC) in the interval 4–6 h post administration, compared with glucose. However, the increase of SCFAs did not affect the plasma concentration of GLP-1 or peptide YY, although a decrease of ghrelin concentration at 6 h after inulin administration was observed [80].

Another study included 60 patients with T2DM that were randomly allocated to four groups: the first group received C4 (as sodium butyrate capsules), the second group received inulin (supplemented as powder), the third group received both C4 and inulin, and the fourth group consumed a placebo for 45 consecutive days. The fasting plasma glucose decreased significantly in the third group and GLP-1 levels were higher in the first and third group compared to the placebo group (suggesting that C4 supplementation can have favorable effect on GLP-1 secretion) [81,82].

The effect of a colonic infusion of SCFAs mixtures (200 mmol/L) high in C2, C3, or C4 versus placebo was studied in a randomized, double-blind, crossover study with the participation of 12 normoglycemic men with overweight or obesity. All SCFAs infusions increased fasting fat oxidation, increased fasting and postprandial plasmatic levels of peptide YY, and decreased fasting free glycerol concentration. Colonic administration of C2 and C4 also significantly increased resting energy expenditure compared to placebo [40]. All these effects have the potential to improve insulin resistance and promote weight loss in T2DM patients with obesity, but to this day, we do not have long-term human studies.

FFAR2 and FFAR3 are also present in beta-cells, with potential direct effect of SCFAs on insulin secretion [83]. An in vitro study performed on isolated islets of Langerhans from non-diabetic human donors showed that the effect of stimulating insulin secretion of SCFAs (C2 and C3) is mediated through FFAR2. In the same study, it was reported that through the same receptor, C2 and C3 have a protective effect against islet apoptosis [84]. However, there are also conflicting results regarding SCFAs’ effect on the beta-cells. The expression of the FFAR2 and the plasma concentration of C2 are increased with a high-fat diet. FFAR2-/- mice fed a high fat diet displayed reduced beta-cell mass and in vitro treatment of isolated human islets with a specific agonist of FFAR2 increased insulin secretion, making FFAR2 a potential therapeutic target for T2DM [85]. On the other hand, double deletion on FFAR2 and FFAR3 (whole body or at pancreatic level) leads to greater insulin secretion and improvement of glucose tolerance in obese, T2DM mice fed with high fat diet compared to controls, but with no effect on glucose control if the deletion of the receptors was in the intestinal cells. The authors concluded that under diabetic condition, the effect of C2 mediated through FFAR2 and FFAR3 is to inhibit insulin secretion stimulated by hyperglycemia [86].

Due to the presence of FFAR2 and FFAR3 in the immune cells, agonists like C2, C3, and C4 can reach plasma levels to stimulate these receptors and play a role in immunogenic response. C2 has fast tissue extraction from the plasma and can hardly reach very high levels; therefore, it probably has little pathological effect on the activity of the immune cells. However, C3 can accumulate in the blood, especially in extreme pathological conditions, like propionic acidemia, with severe impairment of immune function [66]. An interesting experiment conducted by Marino E et al. [87] showed that in mice of non-obese diabetic strain (NOD mice), the use of a high-amylose maize starch diet enriched with C2, C4, or both decreases the occurrence of T1DM versus a high-amylose maize starch diet, normal diet, or high-amylose maize starch diet enriched with C3. The beneficial effects of enriched diets were to modulate autoreactivity mediated through T cells, enhancing gut integrity and decreasing plasma concentration of IL-21 (a known diabetogenic cytokine). A recent published study investigating the effect of high-amylose maize-resistant starch diet enriched with C2 and C4 administered for 6 weeks in patients with long standing T1DM was shown to improve the immune function up to 12 weeks, together with increased stool and plasma concentration of SCFAs. There was no change in HbA1c, glycemic parameters recorded on continuous glucose monitoring systems, insulin doses, food intake, or weight from baseline to week 6 or 12 [88].

In GDM, a gut dysbiosis was observed, with decreased SCFA-producing bacteria, but trends of the alterations for some bacteria are inconsistent. For example, although some bacteria are known to have beneficial effect by the production of SCFAs, they may also produce other proinflammatory molecules with negative impact on glycemic metabolism (such as Bacteroidetes that produce also gram-negative lipopolysaccharide) [89].

### 3.2. Effect of Clinical Management of Diabetes Mellitus on SCFAs Production

The first step in clinical management of DM is lifestyle optimization: healthy diet, daily physical activity, non-smoking status, none or moderate alcohol consumption, good quality and duration of sleep, and stress management. Dietary recommendations focus on providing the right amount of macro- and micronutrients to fulfill daily needs, but also to avoid postprandial hyperglycemia and to decrease cardiovascular risk. In recent years, the role of DFs has been studied, and its effects on the physiology and pathophysiology seem to be mediated by SCFAs produced by bacterial fermentation [90]. 

Pharmacotherapy is prescribed according to the type of DM. Insulin therapy is indicated for survival in T1DM and some cases of specific type of diabetes (e.g., pancreatectomy for pancreatic tumors) and for better glycemic control in T2DM, GDM and other forms of specific type of diabetes. In the pharmacotherapy of T2DM, beside insulin, there are many other classes of drugs that have received approval for use: biguanides, sulphonylureas, glinides, thiazolidindiones, alpha-glucosidase inhibitors, dipeptidyl peptidase-4 (DPP-4) inhibitors, glucagon-like peptide 1 (GLP-1) analogs/GLP-1 receptor agonists, sodium-glucose–linked transporter 2 (SGLT-2) inhibitors [91], and recently approved, dual gastric inhibitory polypeptide (GIP)/GLP-1 receptor agonist [92].

Metformin is commonly used in the pharmacotherapy of T2DM patients. Its beneficial effects can be explained by three major mechanisms of action: first, it inhibits gluconeogenesis and glycogenolysis in the liver; second, it improves insulin sensitivity and glucose uptake in the muscles and peripheric tissues; and third, it delays the intestinal absorption of glucose [93]. In more than 10% of users, at the beginning of the treatment, metformin can cause adverse gastrointestinal effects such as diarrhea, nausea, vomiting, abdominal pain, and loss of appetite [93]. Part of these gastrointestinal side effects can be prevented by gradual increase of dose and there are some evidences that the use of a gastrointestinal microbiome modulator can have beneficial effects on tolerability. The composition of the gastrointestinal microbiome modulator was: inulin from agave, beta-glucan from oats, and polyphenols from blueberry pomace) [94]. Metformin can impact SCFAs production by interaction with host microbiota. In a comparison between nine non-users of metformin versus fifteen users (all T2DM patients), it was observed that SCFAs levels in metformin-users was significantly higher, especially for C3 [95].

The impact of dapagliflozin (a SGLT-2 inhibitor) or gliclazide (a sulphonylurea) as an add-on to metformin on microbiota of 44 T2DM patients was studied over 12 weeks of therapy. The results showed no changes to the fecal microbiome, and the conclusion was that beneficial glycemic effects of these two drugs were not mediated through microbiota [96]. 

Although there are some evidences from animal studies that thiazolidindiones can have a mild effect on gut microbiota, there are no available data from human studies regarding the effect of thiazolidindiones on human microbiome and SCFAs production [97].

Studies on animal models showed that alpha-glucosidase inhibitors have the potential to modify gut microbiota in a reversible and diet-dependent manner, with increased SCFAs production (especially C4) [98]. The role of alpha-glucosidase inhibitors on modulating human microbiota is under evaluation. It was observed that beyond the beneficial therapeutic effect in T2DM by inhibition of human alpha-glucosidase and reduced absorption of dietary carbohydrates, the drug can also inhibit gut bacteria alpha-glucosidase, with an impact on bacterial ability to metabolize carbohydrates, and can be a cause of fluctuation in the structure of human microbiota [99]. On the other hand, it was recently reported that human gut bacteria encode resistance to acarbose [100].

GLP-1 is an incretin hormone secreted by the intestinal L-cells, with beneficial effects in DM by several mechanism of actions, such as lowering plasma glucose value, increasing insulin-secretion dependent on glycemia, and preserving beta-cell mass. GLP-1 analogs are used to treat T2DM, with proven benefits for better glycemic, weight loss, and cardiovascular protection [91]. Although there are data about the relation between SCFAs production and increased endogen secretion of GLP-1 (to be discussed in the prior section), there is no clear evidence that pharmacotherapy with GLP-1 analogues in humans with T2DM modifies production of SCFAs. A change in the microbiome of mice with diet-induced obesity treated with GLP-1 RA liraglutide (0.2 mg/kg, BID) and dual GLP-1/GLP-2 receptor agonist (GUB09–145, 0.04 mg/kg, BID) was observed, with potential to influence SCFAs production [101]. A recent published study enrolled 51 T2DM patients treated with metformin and/or sulphonylureas. They were randomized to receive either 1.8 mg s.c. of liraglutide (a GLP-1 analogue) or sitagliptin (a DPP4-inhibitor), or placebo, daily, for 12 weeks. At the end of the study, no change of gut microbiota was detected [101].

## 4. Conclusions

The role of SCFAs in DM remains to be elucidated, because there are conflicting results regarding the effect of different SCFAs (C2, C3, or C4), current data suggesting potential effects via other metabolic paths than through specific receptors and with different responses in pathological versus physiological conditions. It is not well established how to supplement SCFAs: via modulation of gut SCFAs-producers through special dietary intervention, administration of prebiotics, or direct administration of specific SCFAs. Additionally, is not yet clear which route of administration and what doses should be recommended in clinical practice. The potential role of SCFAs in T1DM is based on evidence suggesting a beneficial immunomodulator effect for C2 and C4, and a neutral (or even negative) effect of C3. In GDM, a modification of SCFAs circulatory levels has also been observed, but it remains to be established what SCFAs, which route of administration, and what concentration can have therapeutic effect in humans.

## Figures and Tables

**Table 1:** Main functions of Enteric Nervous System and their influencing factors. Abbreviations: CNS, Central Nervous System; ICC, interstitial cells of Cajal; EECs, Enteroendocrine Cells; IPAN, intrinsic primary afferent neurons.

The Function of the ENS	The Component of the ENS Involved	Reference	Factors That Influence ENS Activity
Gut motility	Myenteric plexus ICCs-phasic contractions SIP syncytium	[19]	Diet composition Vit D supplementation Antibiotics CNS disorders [25,26,27,28,29]
Secretion and absorption of nutrients	Submucosal plexus IPANs	[19]	
Integration of intrinsic and extrinsic mediation	Intrinsic sensory neurons- modulated by molecules secreted by the EECs Sympathetic and vagal afferent nerves	[23]	
Vasodilation of submucosal arterioles and consecutive hyperemia	IPANs Mechanosensitive enteric neurons	[24]	
Epithelial proliferation, differentiation, and repair	Enteric neurons	[21]	
Modulation of epithelial barrier	Enteric neurons Enteric glial cells	[21]	
Intestinal immune modulation	Cholinergic anti-inflammatory pathway (CAIP)	[21,30]	
Metabolism mediation	Enteric neurons via enterosynes	[29]	

## Data Availability

Not applicable.
